# Involving patients with multimorbidity in service planning: perspectives on continuity and care coordination

**DOI:** 10.15256/joc.2016.6.81

**Published:** 2016-09-16

**Authors:** Michaela L. Schiøtz, Dorte Høst, Anne Frølich

**Affiliations:** ^1^Research Unit for Chronic Conditions, Bispebjerg University Hospital, Copenhagen, Denmark

**Keywords:** Comorbidity, integrated care, multimorbidity, patient satisfaction, qualitative research, user involvement

## Abstract

**Background:**

The prevalence of multiple comorbid chronic conditions, or multimorbidity, is increasing. Care provided to people with multimorbidity is often fragmented, incomplete, inefficient, and ineffective. As part of a research and development project focusing on improving care, we sought to involve patients with multimorbidity in the planning process.

**Objective:**

To identify opportunities for improving care by understanding how patients from a Danish University Hospital experience care coordination.

**Design:**

Qualitative semi-structured interviews with 14 patients with multimorbidity.

**Results:**

Patients with multimorbidity described important concerns about care that included: (1) disease-centered, rather than patient-centered, care; (2) lack of attention to comorbidities and patient preferences and needs; and (3) involvement of numerous healthcare providers with limited care coordination. Poor continuity of care resulted in lack of treatment for complex problems, such as pain and mental health issues, medication errors, adverse events, and a feeling of being lost in the system. Receiving care from generalists (e.g. general practitioners and healthcare professionals at prevention centers) and having a care coordinator seemed to improve patients’ experience of continuity and coordination of care. Suggestions for service improvements when providing care for people with multimorbidity included using care coordinators, longer consultation times, consultations specifically addressing follow-up on prescribed medications, and shifting the focus of care from disease states to patients’ overall health status.

**Conclusions:**

A need exists for a reorganization of care delivery for people with multimorbidity that focuses on improved care coordination and puts patient preferences at the center of care.

## Introduction

The prevalence of multimorbidity, commonly defined as the presence of two or more chronic medical conditions, is rising and rapidly increasing as the population ages [1]. Multimorbidity is associated with decreased quality of life, functional decline, and increased healthcare utilization [2–6]. Patients with multimorbidity have a high treatment burden in terms of understanding and self-managing their conditions, attending multiple appointments, and managing complex drug regimens [7]. Prior studies show that patients often receive care that is “fragmented, incomplete, inefficient and ineffective” [8–12] and that coordination and continuity of care are lacking when several healthcare providers are involved in the treatment of multiple conditions [13]. Thus, efforts seeking to improve coordination and continuity of care have a significant potential impact on the treatment burden experienced by patients with multimorbidity. Three dimensions of continuity are typically described in the literature: information, management, and relational continuity [14]. When all three dimensions are in place, patients experience predictability, safety, and continuity of care.

Smith *et al.* suggest that planning interventions aimed at people with multimorbidity should include the perspectives of patients and their relatives [15]. The benefits of involving users in service planning include potential improvements in services because patients, especially those with long-term illnesses, often have insights into their care that healthcare providers and policymakers lack; in addition, users may not have the same conflicts of interest as healthcare professionals and policymakers [16]. Furthermore, according to Nilsen *et al*., involving service users can lead to more accessible and acceptable health services [16].

The objective of this study, which was part of a larger research project aimed at improving care pathways for people with multimorbidity, was to understand how patients with multimorbidity from the Bispebjerg University Hospital experience continuity and care coordination, and to identify opportunities for improving care in the Danish Capital Region.

## Materials and methods

We conducted individual semi-structured qualitative interviews with patients with multimorbidity from the Bispebjerg University Hospital. Two researchers (M.L.S. and D.H.) conducted all interviews using an interview guide informed in part by concepts of continuity of care derived from Haggerty *et al.* [14], as well as challenges relating to caring for individuals with multimorbidity identified in the literature. We asked patients to describe (1) their care pathways during the last 2 years, and (2) areas of their care that had worked well and those that had not worked well. In addition, we asked about information sharing among healthcare providers, access to services, care transfers within the healthcare system, long-standing relationships with one or more healthcare provider, and the roles of patient, family, and significant others in care management and coordination. Finally, we asked patients for their suggestions on how to improve the service. The interview guide was revised after feedback from other researchers and pilot testing with two participants.

### Setting and participants

Interviews were conducted with patients with multimorbidity who were receiving care at the Bispebjerg University Hospital, operated by the Capital Region as part of the Danish Healthcare System (DHS). A publicly funded healthcare system, which is comparable to the healthcare systems in other Scandinavian countries and the UK [17, 18]. In the DHS, people with chronic conditions are primarily treated by general practitioners (GPs) who can refer patients with complicated needs or progressive disease to hospital-based specialists. Patients can also be referred for rehabilitation to prevention centers run by municipalities or to rehabilitation programs at hospitals. The rehabilitation programs include physical training, dietary advice, and education about specific diseases and medications.

Data from the Capital Region showed that the most prevalent forms of multimorbidity included co-occurring chronic obstructive pulmonary disease (COPD), heart disease, diabetes, and/or depression (source: Region Hovedstaden, internal data not shown). Consequently, we identified people aged 18 years or older with COPD and heart disease, COPD and diabetes, COPD and depression, heart disease and diabetes, heart disease and depression, or diabetes and depression who had been hospitalized or had experienced one or more outpatient clinic visits in 2013. We identified patients using administrative data from the Bispebjerg University Hospital, as the researchers were employed at the hospital and thereby had access to the system. Patients with dementia, mental instability, or the inability to understand or speak Danish were excluded from the study. To ensure that a range of perspectives was included, even though all patients received care at a single hospital, we used purposeful sampling to maximize variation among participants in terms of age, gender, and ethnicity (i.e. native and non-native Danes). Initially, 18 participants were identified and invited to participate in interviews. Fourteen (78%) chose to do so; the remaining invitees (*n*=4, 22%) declined or were too ill to participate.

Each participant received an informational letter about the study and the interview process and signed a written informed consent to participate and to allow us to access their health records. We obtained information about diagnoses and the number of hospitalizations from the health records. Approval to conduct the study was obtained from the Danish Data Protection Agency.

Semi-structured interviews were conducted in the participants’ place of residence (home or nursing home), in a meeting room at the hospital, or over the phone (one interview), according to participants’ preferences. Each interview lasted 45–60 minutes, and all interviews were audio recorded and transcribed verbatim.

### Analysis

All transcribed interviews were coded and categorized inductively using manifest qualitative content analysis [19–21], primarily inspired by Graneheim and Lundman [19]. Firstly, two authors (M.L.S. and D.H.) read the transcribed interviews to obtain a sense of the whole content [19, 20]. Secondly, using an inductive approach to data and Nvivo 10 software, the text was sorted into units of meaning that were then condensed and grouped to create categories and sub-categories [19]. These are listed in Table 1. To introduce rigor and ensure trustworthiness and credibility, categories and sub-categories were first generated individually by two authors (M.L.S. and D.H.), after which, the categories and sub-categories were compared and discussed by all authors and an external researcher with expertise in chronic care management. To create categories that were internally homogenous and externally heterogeneous, the content of categories and sub-categories was compared with that of other emerging categories to ensure that data in categories belonged together and not to other categories [19, 20]. Finally, descriptions and concepts were summarized into main findings, which were discussed by all authors until agreement was reached. To ensure that no meaning was lost in the process, every transcribed quotation was analyzed and discussed in detail with the authors.

Two authors (M.L.S. and D.H.) reviewed participants’ health records to obtain information about diagnoses and number of hospitalizations. The reviews were compared, and in case of a disagreement, the records were reviewed a second time.

## Results

The mean age of the participants, who consisted of four men and ten women, was 71.3 years (range 49–88 years). Nine participants lived alone, one lived in a nursing home, and four lived with a partner. Participants had an average of 3.4 diagnoses (range 2–5), and four had a recorded diagnosis of mental illness. Participants had an average of 2.4 hospitalizations (range 0–6) in the year before the study (Table 2). Several hospitalizations lasted for more than a week, and some lasted for more than 4 weeks. In addition, participants were characterized by reduced functional capacity; most were walking-impaired and used Zimmer frames or electric wheelchairs. Finally, the majority of participants reported feeling lonely and having narrow social networks.

Four categories reflecting multiple opportunities to improve care emerged from the analysis of the interviews (Table 1). “Overall experience of care” included two sub-categories related to describing received care as satisfactory and describing poor experiences of care. “Focus of care” included sub-categories related to whether the participants felt they were treated as whole persons with multiple conditions and individual needs; how the limited time with healthcare providers influenced experiences and outcomes; and how the specialization of care and lack of cross-specialty treatment affected the treatment process. “Medication management” included sub-categories related to medication reconciliation and sharing of information related to medication across providers. “Care coordination” included sub-categories related to the multiple numbers of providers involved in care pathways, how follow-up was experienced, how care was coordinated by physicians and patients, and how participants experienced lack of care coordination leading to adverse events. In addition, the analysis revealed several areas for service improvements suggested by patients, which are presented with the related categories.

### Overall experience of care

In general, the analysis revealed a dichotomy in the way participants described their care experiences. The same participants not only described several areas in the care process that did not work or could be improved but also said that they were generally satisfied with and grateful for their care, a sentiment that was often expressed at the end of the interviews. Statements about overall satisfaction with care seemed to be a way for participants to balance or soften previous criticism about their experiences. As one participant said, “*In general I have to say, except for the last time when I must have been under a bad star, they [the healthcare professionals] have been very competent and nice and most of the time there was a physician coming to me and explaining in detail what I should do and what I should avoid… So in general I am happy about it…*” (*Participant 1*).

### Focus of care

Participants reported that they felt that they were not treated and perceived as whole persons with a complex state of health that included multiple pathologies. They attributed this to the specialization of hospital care and overall lack of time and resources in the healthcare system. Many participants expressed not feeling understood as whole persons with multiple clinical problems and personal issues. Some patients reported that they experienced healthcare professionals as only having the capacity or time to deal with a single problem. Participants reported feeling nervous because none of the many healthcare professionals involved in their care seemed to have a complete understanding of their treatment or the capacity to assess whether ongoing treatment was appropriate or required modification. As one participant reported, “*… I think you are very much one condition and one condition. But you are several conditions, right? Sometimes I think that it is a bit irritating that you are not [perceived] as a whole person… No one has been able to put it all together and make it come together. I haven’t felt that…*” (*Participant 8*).

Similarly, participants felt that their care was not based on their preferences and needs, and that the focus was often on specific conditions and not on more complex or diffuse issues, including mental health problems. They described issues that they felt no one took care of. For example, if they came into the hospital with a problem that was not easy to fix, they were discharged without anyone taking care of it. One participant described it in this way, “*…So you go there and struggle with this [thick swollen legs] all the time and I don’t know why I get these swollen legs. And no one really takes care of it. [‘Oh, we don’t know either’]. Well, so it just has to disappear…*” (*Participant 12*).

Participants felt that the division of care into specialized areas meant that hospital specialists focused on their specialty area and neglected problems or symptoms related to other clinical areas or more general problems such as pain, incontinence, and vertigo. Furthermore, participants believed that clinicians discussed cross-disciplinary issues with providers from other specialties to only a limited extent.

One participant described the healthcare professionals at specialty units as “*lacking knowledge*” about how medical treatment for the condition they were addressing would impact a patient’s other conditions. He suggested that disease-specific departments could consult with medical specialists from other relevant departments, as needed. For example, if a person with diabetes was hospitalized for a COPD exacerbation, the pulmonologist from the respiratory medicine department could contact an endocrinologist to ensure that care was aligned with the patient’s diabetes treatment plan.

Conversely, some participants reported that their GP took their entire health picture into account when providing care. Similarly, participants who had also been seen at a municipal prevention center reported that they felt that healthcare providers, who were typically nurses or physiotherapists, at the center provided care that embraced their full health status, including mental health problems and personal circumstances. Several participants suggested that healthcare professionals at prevention centers were allocated more time to provide care to people with several conditions and complex illness courses.

### Medication management

Participants reported having several healthcare providers who prescribed medications; however, no healthcare provider checked whether the patient’s medication regimen required adjustment because of polypharmacy.

Some participants managed their medications with assistance, for example, filling up their pillboxes on alternate weeks; others had a home nurse help with medication every day. Some patients described not feeling confident that the home nurses had the right information to dispense the right medication and described not being listened to when they raised concerns about the medication that was provided.

Participants who had a GP or a specialist taking an active role in coordinating their care expressed confidence that their medications were appropriate. Conversely, patients who did not have a healthcare professional coordinating their care reported feeling unsure about whether the medication they received was appropriate. As one patient described, “*I get something preventive to the heart, right? I get anticoagulant and some preventive pills and some cholesterol pills. And then I have one of these glycerins there, which I actually don’t use. Then I have also had depression and for that I get these and these. And this one is gastric acid. This is the one the hormone doctor gave to me, which I don’t need anyway. And these I get for the pain. But I only take them if I know I have to walk. Sometimes I think, ‘Is this treatment, is it preventive, or is it pain-relieving?’ I am sometimes in doubt about that… And then I also get a hormone supplement…*” (*Participant 8*).

One participant suggested that consultations aimed at following up on medications, including assessing the need for discontinuation, would be valuable.

### Care coordination

Participants described seeing many different healthcare providers, many of whom were new to them. The involvement of numerous healthcare providers affected care in several ways. Some participants described feeling left alone to manage their own care, despite the involvement of many healthcare providers. As one participant explained, “*A professor and a chief physician are involved in this, and still I think I am very much left on my own with it*” (*Participant 8*).

Other participants expressed feeling exhausted from having to deal with new people all the time and described information being lost when new healthcare providers became involved in their care, which sometimes resulted in medical errors. They described medical errors that included failure to prescribe vital medicine that led to withdrawal symptoms, lack of wound management leading to hospitalization, and failure to provide essential medications during hospitalizations.

When participants felt that their GP or their hospital specialist was following up regularly, they also felt that their care was under control. Some participants described their GP or one of the medical specialists involved in their treatment as taking an active role in coordinating their care. They described the GP or hospital specialist following up on their treatment or contacting them if they did not keep a scheduled appointment. One participant described her GP’s involvement as, “*I feel that she takes really good care of me now. It’s like she calls and asks how I am doing almost without me arranging it. So that is really pleasant…*” (*Participant 12*).

However, most participants reported that no healthcare professional coordinated their care. A participant expressed the lack of a care coordinator by saying, “*… So I haven’t had a steadfast person you can say is responsible – not at all*” (*Participant 3*).

Some participants described their GPs as following their care without being actively involved in coordinating it. Participants described that they needed to coordinate their care because no one else was doing it. Some participants described feeling powerless when they tried to deal with the healthcare system on their own. For example, one participant reported not receiving eye drops for glaucoma during a week-long hospitalization, even though she had asked for them several times. In addition, participants described receiving conflicting information from different healthcare providers. As one participant said, “*Every time you come, then one doctor says, ‘Yes, you have a giant infection or you have a lot of infection’. Then the next day another doctor comes and says, ‘Well, this is not something we need to treat…*’” (*Participant 12*).

This led to the suggestion that future care should include a person responsible for coordinating care for people with multiple conditions. As one participant described it, “*…It is also a lot to take all these confrontations with one and then the other and wait and wait and wait… When will something happen, right? Instead of one person there are a lot of people you have to talk to and you cannot get in contact with them… Instead, you could have one contact person that knows more about how you were doing and how you can be helped in the best way. But you are just thrown around to 100 different places…*” (*Participant 12*).

## Discussion

Our findings portray a system in which numerous healthcare professionals make disjointed efforts to provide care that may be fruitless, unless a healthcare professional focuses on the overall health status of the patient and coordinates the efforts of all involved providers. Participants did not feel that they were cared for as whole persons. Instead, numerous healthcare professionals focused on different organ systems, but no one coordinated their efforts, took patients’ personal preferences into account, or addressed non-urgent problems. Additionally, participants experienced medical errors caused by a lack of both care coordination and attention to comorbidities and patient concerns. The exceptions to this were patients who identified a healthcare professional as actively assuming responsibility for care coordination. Participants receiving care from generalist care providers (GPs or healthcare professionals in municipal prevention centers) experienced a more patient-centered approach to care and felt more confident about and satisfied with their care.

Our findings highlight the magnitude of problems related to care for people with multimorbidity and also suggest improvements. The existing organization of care may lead to suboptimal care and medical errors, and there is a need for a reorganization of healthcare delivery. Our findings suggest that opportunities for specific improvements include assigning responsibility for care coordination for people with multimorbidity, providing longer consultation times, shifting the focus of care from disease states to patients’ overall health status, and ensuring that patients are cared for by the right group of healthcare professionals, e.g. generalists versus specialists.

Our results are consistent with those of other studies reporting quality of care deficiencies when patients have discordant or unrelated comorbidities [22–26]. The experiences participants described of feeling exhausted and left to manage care on their own are consistent with findings from studies in other healthcare systems comparable to the DHS. In a qualitative study of patient experiences across the interface between primary and secondary healthcare in England, Preston *et al.* found that patients were not provided with sufficient support, information, and continuity of care, leading to feelings of helplessness and insecurity, and making progress through the system very difficult [27]. In a Swedish qualitative study focusing on experiences of continuity of care when patients saw multiple clinicians, Von Bultzingslowen *et al.* found that having a single trusted clinician who helped navigate the system and saw the patient as a partner, provided a foundation for continuity of care between clinicians [28]. Similarly, a qualitative study conducted in the UK found that relational continuity was a valuable resource for preventing and repairing gaps in care [29]. This is in accordance with our finding that patients who had a physician coordinating their care felt confident and secure about their care. Likewise, other studies have shown that patients associate good relationships with healthcare professionals with the experience of progress, expecting and experiencing that their GP or specialist will take responsibility if treatment stalls or is misdirected [28, 30]. Studies among patients with lifelong disease courses who are often being treated for several diseases that require ongoing contact with different parts of the healthcare system identify that experiences of continuity of care are often linked to relational continuity [30, 31].

The results of our study also revealed that participants experienced that the limited time available at consultations affected the quality of care and the ability of healthcare professionals to provide comprehensive care. This is consistent with other studies identifying lack of time as a barrier to providing care for people with multimorbidity [32–34]. Furthermore, the evidence suggests that additional time spent in consultations leads to more preventive health advice, less prescribing, and increased patient satisfaction [34].

Although participants described experiencing several problematic incidents reflecting inadequate and uncoordinated care, they also reported being generally satisfied with their care. This paradox may be explained by the dramaturgical model of social interaction [35]. Patients may have an outward or “front stage” appearance of satisfaction because they are dependent on healthcare professionals, but feel free when they are “back stage” to tell an interviewer who is not a healthcare professional about negative care experiences. Patients may consciously or unconsciously perceive that expressing dissatisfaction is risky. Furthermore, patients may not know where to direct their discontent and, to avoid expressing it, may instead choose to voice gratitude for the system on which they depend, despite its flaws. Investigating this phenomenon is beyond the scope of this study, but future research should examine the relationship between overall patient satisfaction and specific complaints about care.

The strengths of this study include the use of qualitative methods that made it possible to obtain a rich understanding of how people with multimorbidity experience continuity of care and perceive opportunities for improvement. Furthermore, several steps were taken to strengthen the study validity, including an exact description of the study design, extensive author discussion of the findings, and illustrating the findings with quotations from interviews to establish confirmability [36]. The limitations of the study include the recruitment of participants from a single hospital. However, participants also received care from other hospitals in the Capital Region, from their GPs, and from the municipalities. We believe that the findings are representative of the larger group of patients with multimorbidity in the Danish Capital Region. Participants were selected for recruitment based on a combination of a limited number of chronic conditions, but they had several additional diagnoses, including cancer and mental health conditions in addition to depression. Consequently, the patient experiences we report may also represent those of patients with more than the four chronic conditions that we used to select participants. However, participants in our study had an average of 3.4 chronic diagnoses, which is relatively low in comparison with the average number of conditions among people with multimorbidity in other studies [1, 37]. This may indicate that participants in our study represent the “healthier part” of the population of all patients with multimorbidity. This may therefore limit the transferability of our findings to patients with more comorbid conditions, who may have different or more extreme experiences of problematic care, or suggest different care improvements. However, the participants in our study were characterized by significantly reduced functional capacity and several complex issues, which could also indicate that not all diagnoses were listed in the patients’ records, a potential deficit in medical record keeping. Another limitation is the relatively small sample size of participants. Interviewing more patients with multimorbidity may have revealed other experiences or different aspects of the experiences we report. Nevertheless, the findings of our study are consistent with those from other parts of the DHS [29, 31] and other healthcare systems [14, 27, 30, 38–40]. We believe that our findings include central areas of care experiences for adults with multimorbidity.

Using patient experiences to identify improvement opportunities may be a valuable method for investigating whether challenges and solutions identified in other settings are also present in and relevant to the setting in which an intervention or organizational restructuring is being planned. For people with multimorbidity, implementing the use of care coordinators, increasing consultation times, devoting consultations to following up on medications, and changing the focus of care from individual diseases to overall health would likely lead to more accessible and acceptable care. The reasons why these solutions have not already been implemented on a widespread basis are unclear; it is questionable whether including patients’ perspectives in the planning of services will make it more likely that these elements will be included in care programs. Implementation will be influenced by practical, political, and structural circumstances. Our findings suggest that involving patients in service planning can provide a complete picture of the existing situation, which is essential to identifying required changes. Patient experiences can be used as a starting point for planning of care, but more efforts addressing policymaking, change management, and cultural change, are also needed. We note that our findings also emphasize the importance of being aware of implicit or explicit agendas that may influence how patients report their experiences.

Further research is needed to investigate how the healthcare system should be organized to provide patient-focused coordinated care to people with multimorbidity. This includes investigating how care coordination can be ensured, how the number of visits and number of healthcare professionals involved in the care pathway can be reduced, how consultation times can be increased, and how disease-specific specialized care can co-exist with patient-centered generalist care.

## Figures and Tables

**Table 1:** Categories and sub-categories of patient experiences of healthcare.

Categories	Sub-categories
Overall experience of care	Satisfactory care experiencesPoor experiences of care
Focus of care	Treated as a whole person Specialized care Time with healthcare providers Collaboration across specialties
Medication management	Follow-up on medication Information about medication across providers
Care coordination	Many healthcare providers involved in the care pathways Care coordination Follow-up on care Physicians as care coordinators Patients as care coordinators Adverse events

**Table 2:** Characteristics of participants.

Participant number	Gender	Age (years)	Living alone	Chronic conditions	Number of hospitalizations^*^ in 2013
1	Female	85	Yes	Heart disease, apoplexy, COPD, ulcus cruris, glaucoma	3
2	Female	75	Yes	Heart disease, back disease, COPD, diabetes, anxiety	3
3	Female	71	No	Heart disease, apoplexy, diabetes, depression	2
4	Male	69	Yes	Heart disease, COPD, diabetes, nephropathy	0
5	Male	56	No	COPD, diabetes	0
6	Female	75	Yes	Heart disease, COPD	2
7	Female	75	Yes	Heart disease, diabetes, Parkinsonism	6
8	Female	63	Yes	Heart disease, back disease, cancer, depression	2
9	Male	75	No	Heart disease, back disease, COPD, diabetes	1
10	Female	88	Yes	Heart disease, osteoarthritis, depression	1
11	Female	81	Yes	Heart disease, COPD, diabetes	4
12	Female	49	Yes	COPD, diabetes, depression	6
13	Female	83	Yes	Heart disease, COPD, diabetes	1
14	Male	53	No	Heart disease, COPD, diabetes	3

^*^Transfers are not calculated as separate hospitalizations. COPD, chronic obstructive pulmonary disease.
